# Prenatal Exposure to Organophosphorous Pesticides and Fetal Growth: Pooled Results from Four Longitudinal Birth Cohort Studies

**DOI:** 10.1289/ehp.1409362

**Published:** 2015-12-18

**Authors:** Kim G. Harley, Stephanie M. Engel, Michelle G. Vedar, Brenda Eskenazi, Robin M. Whyatt, Bruce P. Lanphear, Asa Bradman, Virginia A. Rauh, Kimberly Yolton, Richard W. Hornung, James G. Wetmur, Jia Chen, Nina T. Holland, Dana Boyd Barr, Frederica P. Perera, Mary S. Wolff

**Affiliations:** 1 Center for Environmental Research and Children’s Health (CERCH), School of Public Health, University of California, Berkeley, Berkeley, California, USA; 2 Department of Epidemiology, UNC Gillings School of Global Public Health, Chapel Hill, North Carolina, USA; 3 Department of Environmental Health Sciences, Columbia Center for Children’s Environmental Health, Mailman School of Public Health, Columbia University, New York, New York, USA; 4 Simon Fraser University, Vancouver, British Columbia, Canada; 5 Department of Population and Family Health, Mailman School of Public Health, Columbia University, New York, New York, USA; 6 Division of General and Community Pediatrics, Cincinnati Children’s Hospital Medical Center, Cincinnati, Ohio, USA; 7 Department of Microbiology, and; 8 Department of Preventive Medicine, Icahn School of Medicine at Mount Sinai, New York, New York, USA; 9 Department of Environmental Health, Rollins School of Public Health, Emory University, Atlanta, Georgia, USA

## Abstract

**Background:**

Organophosphorous (OP) pesticides are associated with reduced fetal growth in animals, but human studies are inconsistent.

**Objectives:**

We pooled data from four cohorts to examine associations of prenatal OP exposure with birth weight (n = 1,169), length (n = 1,152), and head circumference (n = 1,143).

**Methods:**

Data were from the CHAMACOS, HOME, Columbia, and Mount Sinai birth cohorts. Concentrations of three diethyl phosphate (ΣDEP) and three dimethyl phosphate (ΣDMP) metabolites of OP pesticides [summed to six dialkyl phosphates (ΣDAPs)] were measured in maternal urine. Linear regression and mixed-effects models were used to examine associations with birth outcomes.

**Results:**

We found no significant associations of ΣDEP, ΣDMP, or ΣDAPs with birth weight, length, or head circumference overall. However, among non-Hispanic black women, increasing urinary ΣDAP and ΣDMP concentrations were associated with decreased birth length (β = –0.4 cm; 95% CI: –0.9, 0.0 and β = –0.4 cm; 95% CI: –0.8, 0.0, respectively, for each 10-fold increase in metabolite concentration). Among infants with the PON1192RR genotype, ΣDAP and ΣDMP were negatively associated with length (β = –0.4 cm; 95% CI: –0.9, 0.0 and β = –0.5 cm; 95% CI: –0.9, –0.1).

**Conclusions:**

This study confirms previously reported associations of prenatal OP exposure among black women with decreased infant size at birth, but finds no evidence of smaller birth weight, length, or head circumference among whites or Hispanics. Contrary to our hypothesis, we found stronger inverse associations of DAPs and birth outcome in infants with the less susceptible PON1192RR genotype. The large pooled data set facilitated exploration of interactions by race/ethnicity and PON1 genotype, but was limited by differences in study populations.

**Citation:**

Harley KG, Engel SM, Vedar MG, Eskenazi B, Whyatt RM, Lanphear BP, Bradman A, Rauh VA, Yolton K, Hornung RW, Wetmur JG, Chen J, Holland NT, Barr DB, Perera FP, Wolff MS. 2016. Prenatal exposure to organophosphorous pesticides and fetal growth: pooled results from four longitudinal birth cohort studies. Environ Health Perspect 124:1084–1092; http://dx.doi.org/10.1289/ehp.1409362

## Introduction

Organophosphorous (OP) pesticides, a widely used class of insecticides, have been the subject of concern in recent years because of their potential for developmental and neurobehavioral toxicity (Bouchard et al. 2011; Engel et al. 2011; Rauh et al. 2011). Two common OP pesticides, chlorpyrifos and diazinon, were removed from residential pesticide use between 2001 and 2004, largely due to concerns about effects on health (U.S. EPA 2001); however, applications in agriculture continue. Although use of OP pesticides has declined substantially in recent years, they still accounted for 35% of insecticides applied in the United States in 2007, the latest year for which data are available (Grube et al. 2011).

Several OP pesticides have been associated with reduced fetal growth in rodent studies (Breslin et al. 1996; Qiao et al. 2002; Spyker and Avery 1977; Srivastava and Raizada 1996), but studies in humans are inconsistent. Two studies have examined maternal or fetal blood concentrations of OP pesticides (Barr et al. 2010; Whyatt et al. 2004), and four studies have looked at maternal urinary concentrations of OP metabolites (Eskenazi et al. 2004; Rauch et al. 2012; Wang et al. 2012; Wolff et al. 2007). Chlorpyrifos concentrations in umbilical cord blood were inversely associated with birth weight and length in a population of low-income, African-American and Dominican women in New York City recruited before the household ban (Columbia cohort) (Whyatt et al. 2004). However, a similar study conducted after the ban found no associations with birth outcome among pregnant women in New Jersey whose chlorpyrifos concentrations were considerably lower than the Columbia study (Barr et al. 2010). Dialkyl phosphate (DAP) metabolites of OP pesticides in maternal urine were inversely associated with infant head circumference but not birth weight or length in a racially and economically diverse population of women in New York City (Mount Sinai cohort) (Wolff et al. 2007). Prenatal urinary DAP metabolites were associated with shorter length of gestation and nonsignificantly increased birth weight, length, and head circumference in a cohort of low-income predominantly Mexican women in California (Center for the Health Assessment of Mothers and Children of Salinas; CHAMACOS cohort) (Eskenazi et al. 2004). A study of pregnant women in Cincinnati, Ohio (Health Outcomes and Measures of the Environment; HOME study) found that prenatal urinary DAPs were associated with decreased birth weight in African-American mothers and shorter length of gestation in white mothers (Rauch et al. 2012). However, a study in Shanghai, China, found no association between maternal urinary DAPs at time of labor and birth weight or length (Wang et al. 2012).

Inconsistencies in these findings may be attributable to differences in timing of exposure (e.g., early vs. late pregnancy), measurement of OP exposure (e.g., parent compounds in blood vs. nonspecific DAP metabolites in urine), variability in OP mixtures (e.g., chlorpyrifos or diazinon used residentially vs. a wider range of OP compounds used in agriculture), and patterns of exposure (residential vs. agricultural vs. dietary exposure) across studies. Other challenges to integrating these disparate results include the diverse racial/ethnic compositions of these cohorts, which contribute to differing frequencies of key genetic modifiers, among other sources of heightened susceptibility. For example, paraoxonase (PON) is an enzyme that plays a key role in metabolism and detoxification of some OP pesticides (Costa et al. 2008). Paraoxonase levels and efficiency are influenced by several common single nucleotide polymorphisms (SNPs) in the *PON1* gene whose allele frequencies vary significantly in different racial groups (Costa et al. 2003). SNPs at the –108 position in the promoter region of the *PON1* gene appear to influence an individual’s quantity of the paraoxonase enzyme (subjects with *PON1_–108TT_* genotype have approximately 20% less paraoxonase enzyme), whereas SNPs at the 192 position in the coding region affect the catalytic efficiency of that enzyme in detoxifying OP pesticides with *PON_192QQ_* coding for the enzyme with the lowest efficiency (Costa et al. 2003).

To explore relationships between prenatal OP exposure and birth outcomes and potential modification by the *PON1* genotype and race/ethnicity, we pooled data from four previously published longitudinal studies—the CHAMACOS, HOME, Columbia, and Mount Sinai cohorts. We hypothesized that DAP metabolites would be inversely associated with fetal growth, and that this effect would be especially prominent in infants with the susceptible *PON1* genotypes with lower enzyme concentration and catalytic efficiency.

## Methods

The study sample combined participants from cohorts that measured urinary DAP metabolites during pregnancy. DAP concentrations from the CHAMACOS, HOME, and Mount Sinai cohorts have been previously reported (Eskenazi et al. 2004; Rauch et al. 2012; Wolff et al. 2007). DAP concentrations were measured on only a subset of the Columbia cohort and have not been published elsewhere. These four studies were conducted using similar methods, with some key differences (Table 1). The CHAMACOS study enrolled low-income women between 1999 and 2000 through prenatal clinics serving the farmworker population of the agricultural Salinas Valley, California (Eskenazi et al. 2004). Women were enrolled into the HOME study though prenatal clinics in metropolitan Cincinnati between 2003 and 2006 (Rauch et al. 2012). The Columbia cohort enrolled African-American and Dominican pregnant women living in northern Manhattan or the South Bronx between 1998 and 2006 (Perera et al. 2003; Whyatt et al. 2004), and the Mount Sinai cohort enrolled an ethnically diverse population of primiparous women receiving prenatal care at Mount Sinai Hospital in lower Manhattan between 1998 and 2002 (Berkowitz et al. 2004; Wolff et al. 2007). Major differences between the cohorts were the geographic, racial/ethnic, and socioeconomic characteristics of the populations. Mount Sinai was the only cohort limited to primiparous women. Additionally, the CHAMACOS and HOME cohorts enrolled participants before 20 weeks gestation and obtained two urinary DAP measures (early and late pregnancy), whereas Mount Sinai and Columbia obtained a single DAP measure in the third trimester. In all four cohorts, participants were at least 18 years of age and started prenatal care before 26 weeks gestation.

**Table 1:** Study design of the individual cohorts.

Design element	CHAMACOS	HOME	Columbia	Mount Sinai
Study location	Salinas Valley, CA	Cincinnati, OH	New York, NY	New York, NY
Sample size	*n *= 486	*n *= 344	*n *= 314	*n *= 404
Enrollment years	1999–2000	2003–2006	1998–2006	1998–2002
Mean gestational age at enrollment	14.1 weeks	16.1 weeks	33.3 weeks	31.8 weeks
Recruitment sites	Six prenatal clinics serving farmworkers	Seven prenatal clinics in Cincinnati	Prenatal clinics at Harlem and NY Presbyterian hospitals	Prenatal clinic at Mt. Sinai Hospital
Eligibility criteria	≥ 18 years old < 20 weeks gestation English or Spanish speaking Low income (Medicaid eligible)	≥ 18 years old < 19 weeks gestation Living in 5 surrounding counties Living in home built before 1978 HIV negative	18–35 years old African-American or Dominican identity Living in Northern Manhattan or South Bronx at least 1 year First prenatal visit < 20 weeks No tobacco or drug use in pregnancy No chronic medical conditions (HIV, diabetes, hypertension)	≥ 18 years old Primiparous First prenatal visit ≤ 26 weeks No alcohol or drug use in pregnancy Singleton birth No chronic medical conditions (diabetes, hypertension) or pregnancy complications
Race/ethnicity	Mexican	Various	African American and Dominican	Various

For the main analysis, we excluded women with hypertension, diabetes, and other pregnancy complications that might affect fetal growth; twins and multiple births; stillbirths and neonatal deaths; and women providing urine samples that were dilute (< 10 mg/dL creatinine). The pooled data set included 1,235 women with urinary DAP measures during pregnancy and birth outcome data from each cohort: 484 women from the CHAMACOS study, 328 women from the HOME study, 82 women from Columbia, and 341 women from Mount Sinai. Informed consent was obtained from all women, and study procedures were approved by the institutional review boards at each research institution.

### Data Collection

In each cohort, mothers were interviewed during pregnancy in their preferred language (English or Spanish). Data collected in common across the four cohorts included demographic information (age, marital status, race, ethnicity, country of birth, educational attainment), behavioral factors (smoking, alcohol, drug use during pregnancy), and pregnancy health (parity, prenatal care use, pregnancy complications). Household income was not collected in one cohort (Mount Sinai) and thus could not be included in the pooled data set; because maternal education level was collected in all cohorts, it was used as a proxy for socioeconomic status.

Infant weight, length, and head circumference at birth were abstracted from medical records at each location. Gestational age at birth was calculated by maternal report of last menstrual period (HOME study) or abstracted from medical records (CHAMACOS, Columbia, Mount Sinai). Because Columbia and Mount Sinai measured OP exposure in a late stage of pregnancy, there was a truncated range in gestational age outcomes and few preterm births in these cohorts, limiting power to detect associations with gestational duration. Thus, gestational age at birth was examined as a covariate in the models of fetal growth, but not as an outcome in the pooled analyses.

### Organophosphorous Pesticide Exposure

In each cohort, spot urine samples were collected from pregnant women at the time of interview. This included two urine samples from women in the CHAMACOS (mean, 13.9 and 26.5 weeks gestation) and HOME (mean, 15.9 and 26.4 weeks gestation) cohorts and one urine collection from women in the Columbia (mean, 33.3 weeks gestation) and Mount Sinai (mean, 31.8 weeks gestation) cohorts. All urinary DAP measurements were conducted by the same laboratory at the Center for Disease Control and Prevention using gas chromatography–tandem mass spectrometry (GC-MS/MS) and quantified using isotope dilution calibration (Bravo et al. 2002). Limits of detection (LODs) varied among the centers and ranged from 0.1 to 0.7 μg/L (see Table S1). Six individual DAP metabolites were quantified: three dimethyl phosphate (DMP) metabolites (dimethylphosphate, dimethylthiophosphate, dimethyldithiophosphate), derived from OP pesticides such as malathion and dimethoate; and three diethyl phosphate (DEP) metabolites (diethylphosphate, diethylthiophosphate, diethyldithiophosphate), derived from OP pesticides such as chlorpyrifos and diazinon. These DAP metabolites, in units of nanomoles per liter, were summed to yield ΣDMP, ΣDEP, and total ΣDAP for each individual. Many women had concentrations below the LOD for one or more of the metabolites, but only 25 women (2.1%) were below the LOD on all 6 metabolites (see Table S1). In cases where individual metabolite concentrations were below the LOD, the machine-read value was used if available or, if not, a non-zero, random number below the LOD was substituted before the individual DAPs were summed. The random values were selected from all values below the LOD using log-normal probability distributions whose cohort-specific parameters were obtained by maximum likelihood estimation (Lubin et al. 2004). Summed concentrations (ΣDMP, ΣDEP, and ΣDAP) were truncated at 3 standard deviations below the geometric mean of the pooled data set to avoid influentially low values. Creatinine concentrations in urine, measured using commercially available diagnostic enzyme methods, were used to account for urinary dilution. Creatinine-corrected ΣDMP, ΣDEP, and ΣDAP concentrations were log_10_-transformed for analysis. For the Columbia and Mount Sinai cohorts, the results of the single urinary measurements were used; for the HOME and CHAMACOS studies, the two measurements during pregnancy were averaged. The correlations between the two ΣDAP concentrations during pregnancy were 0.16 and 0.25 for CHAMACOS and HOME, respectively.

### *PON1* Genotype

*PON1_192_* and *PON1_–108_* genotyping was conducted separately at each research center using polymerase chain reaction (PCR)–based methods using umbilical cord blood for children and maternal blood. Genotyping methods have been previously published for the CHAMACOS cohort (Holland et al. 2006), the HOME Study (Rauch et al. 2012), and the Mount Sinai cohort (Chen et al. 2003). Mothers and children were genotyped as QQ, QR, or RR for *PON1*_192_ and TT, CT, or TT for *PON1_–108_.* Maternal *PON1_–108_* genotype was not available from the HOME study, and maternal *PON1_192_* genotype was not available from the HOME or Columbia cohorts.

### Data Analysis

We first compared demographic characteristics and urinary metabolite concentrations across the four cohorts. We examined the associations of urinary ΣDAP, ΣDEP, and ΣDMP concentrations with infant birth weight, length, and head circumference in the pooled data set using multivariable linear regression. We evaluated effect modification by cohort and performed analyses adjusted for and stratified by cohort. We fit mixed-effects models with random slope and intercept, allowing for different associations in each cohort, and with random intercept only. Mixed-effects model estimates for associations between DAP metabolites and the outcomes were similar to estimates from models that did not include random effects for cohort (data not shown), and mixed-effects models did not have significantly better performance based on likelihood ratio tests (α = 0.05), so estimates from mixed-effects models are not reported.

Creatinine-corrected, urinary DAP metabolites were considered as continuous (log_10_ transformed) variables. All models controlled for cohort, sex, self-identified race/ethnicity [non-Hispanic white, non-Hispanic black, Hispanic (regardless of race), and other], country of origin (USA vs. other), maternal education (< high school vs. ≥ high school), parity (primiparous vs. multiparous), smoking during pregnancy (yes vs. no), marital status (married/living together vs. other), and maternal age (continuous) (Table 2). Covariate information was missing for 66 women, reducing the final sample size to 1,169 for birth weight, 1,152 for length, and 1,143 for head circumference models. The Hispanic category included women of Mexican, Puerto Rican, Dominican, and other Hispanic descent. To examine fetal growth independent of gestational age, we controlled for gestational age at birth using a cubic spline. We tested for effect modification by race/ethnicity, sex, maternal education, and child *PON1_192_* and *PON1_–108_* genotype by including cross-product interaction terms (e.g. for effect modification of DAPs by the 4-level race/ethnicity variable, we constructed 3 cross-product terms: black × DAPs, Hispanic × DAPs, other × DAPs). We compared models with and without the cross-product terms using Wald tests to examine overall categories of each potential modifier rather than category-by-category tests of interaction. We considered interaction to be statistically significant at α = 0.1. Stratified analyses were conducted according to cohort, race/ethnicity, and maternal and child *PON1* genotype using separate models to obtain stratum-specific estimates. Because of the small size, the race/ethnicity category of “other” was omitted from stratified models, although it was included in the models with interaction terms. *PON1* genotype was the most commonly missing variable, so the sample size was reduced for these interaction models (*n* = 947–953 for infant and *n* = 736–804 for maternal genotype).

**Table 2:** Participant characteristics of the individual cohorts and pooled data set.

Characteristic	Pooled		CHAMACOS		HOME		Columbia		Mount Sinai		*p*-Value^*a*^
	*n*	Mean ± SD or %	*n*	Mean ± SD or %	*n*	Mean ± SD or %	*n*	Mean ± SD or %	*n*	Mean ± SD or %	
Gestational age (weeks)	1,235	39.1 ± 1.6	484	38.9 ± 1.7	328	39.2 ± 1.5	82	39.4 ± 1.3	341	39.3 ± 1.5	< 0.01
Birth weight (g)	1,235	3385.0 ± 514.1	484	3449.7 ± 516.4	328	3407.0 ± 566.3	82	3417.0 ± 470.9	341	3264.1 ± 444.9	< 0.01
Length (cm)	1,218	50.6 ± 2.6	478	50.2 ± 2.7	321	51.0 ± 2.8	80	50.9 ± 2.4	339	50.7 ± 2.3	< 0.01
Head circumference (cm)	1,209	34.0 ± 1.6	468	34.1 ± 1.5	322	34.3 ± 1.8	79	34.0 ± 1.5	340	33.8 ± 1.6	< 0.01
Maternal age (years)	1,235	25.9 ± 5.9	484	25.9 ± 5.0	328	28.8 ± 5.6	82	24.1 ± 5.1	341	23.4 ± 6.2	< 0.01
Prepregnancy BMI (kg/m^2^)	1,215	25.6 ± 5.5	484	26.9 ± 5.1	313	26.0 ± 6.3	77	26.0 ± 5.8	341	23.3 ± 4.4	< 0.01
Pregnancy weight gain (lb)	1,186	32.3 ± 15.3	481	30.0 ± 12.4	324	27.1 ± 13.7	78	37.0 ± 16.9	303	40.2 ± 17.1	< 0.01
Child sex
Male	617	50.0	243	50.2	149	45.4	34	41.5	191	56.0	0.02
Female	618	50.0	241	49.8	179	54.6	48	58.5	150	44.0
Year of birth
1998	60	4.9	0	0.0	0	0.0	0	0.0	60	17.6	< 0.01
1999	123	10.0	0	0.0	0	0.0	0	0.0	123	36.1
2000	466	37.7	308	63.6	0	0.0	35	42.7	123	36.1
2001	258	20.9	176	36.4	0	0.0	47	57.3	35	10.3
2003	26	2.1	0	0.0	26	7.9	0	0.0	0	0.0
2004	129	10.4	0	0.0	129	39.3	0	0.0	0	0.0
2005	122	9.9	0	0.0	122	37.2	0	0.0	0	0.0
2006	51	4.1	0	0.0	51	15.5	0	0.0	0	0.0
Maternal race/ethnicity
Non-Hispanic white	288	23.4	8	1.7	210	64.4	0	0.0	70	20.5	< 0.01
Non-Hispanic black	220	17.8	0	0.0	94	28.8	35	42.7	91	26.7
Hispanic	693	56.2	463	95.7	9	2.8	47	57.3	174	51.0
Other	32	2.6	13	2.7	13	4.0	0	0.0	6	1.8
Maternal country of origin
Other	535	45.0	416	86.0	18	5.5	37	45.1	64	21.6	< 0.01
USA	653	55.0	68	14.0	308	94.5	45	54.9	232	78.4
Maternal education
Less than high school	546	44.3	381	78.7	35	10.7	30	36.6	100	29.4	< 0.01
Completed high school	686	55.7	103	21.3	291	89.3	52	63.4	240	70.6
Marital status
Single	382	31.0	96	19.8	65	19.9	64	78.1	157	46.0	< 0.01
Married/living together	851	69.0	388	80.2	261	80.1	18	22.0	184	54.0
Parity
Nulliparous	693	56.2	162	33.5	143	43.9	47	57.3	341	100.0	< 0.01
Multiparous	540	43.8	322	66.5	183	56.1	35	42.7	0	0.0
Smoking during pregnancy
No	1,086	89.4	454	93.8	268	87.0	80	97.6	284	83.3	< 0.01
Yes	129	10.6	30	6.2	40	13.0	2	2.44	57	16.7
Child *PON*_*–108*_
TT	167	16.9	76	17.6	52	17.9	14	20.6	25	12.6	< 0.01
CT	453	45.8	224	52.0	113	38.8	32	47.1	84	42.2
CC	369	37.3	131	30.4	126	43.3	22	32.4	90	45.2
Child *PON*_*192*_
QQ	300	30.1	108	24.8	117	39.8	14	20.9	61	30.5	< 0.01
QR	450	45.2	222	51.0	115	39.1	25	37.3	88	44.0
RR	246	24.7	105	24.1	62	21.1	28	41.8	51	25.5
Maternal *PON*_*–108*_
TT	165	19.5	103	23.0		—	14	20.6	48	14.5	< 0.01
CT	374	44.1	218	48.7		—	31	45.6	125	37.7
CC	309	36.4	127	28.3		—	23	33.8	159	47.9
Maternal *PON*_*192*_
QQ	220	28.2	125	27.9		—		—	95	28.6	0.76
QR	364	46.7	206	46.0		—		—	158	47.6
RR	196	25.1	117	26.1		—		—	79	23.8
BMI, body mass index. ^***a***^*p*-Values for differences across cohorts. *F*-tests for continuous variables, chi-square tests for categorical variables.											

After constructing final models, we conducted sensitivity analyses including *a*) limiting analyses to nulliparous women, *b*) limiting to term births, *c*) treating values < LOD as LOD divided by the square root of 2, *d*) classifying women identifying as Hispanic black (e.g. Dominicans) as black rather than Hispanic, *e*) including women with pregnancy complications such as hypertension and diabetes, *f*) using DAP concentrations that were not corrected for creatinine, and *g*) using only one (late pregnancy) DAP measurement from the HOME and CHAMACOS cohorts. All analyses were conducted in Stata 13.

## Results

The four cohorts differed on every characteristic measured, including racial/ethnic composition, country of origin, maternal education, and marital status (Table 2). The Columbia cohort was exclusively African American and Hispanic (Dominican); the CHAMACOS cohort predominantly Hispanic (Mexican); the Mount Sinai cohort racially diverse, but predominantly Hispanic (Puerto Rican); and the HOME study cohort largely white. Educational attainment was lowest in the CHAMACOS cohort and highest in the HOME study (21% vs. 89% completing high school, respectively). More women were married or living with their partner in the CHAMACOS and HOME studies (~ 80%) than in the two New York City studies (54% for Mount Sinai and 22% for Columbia). In the Columbia study, couples were defined as married/living together only if they had lived together at least 7 years, whereas the other studies included all cohabiting couples. Concentrations of urinary metabolites of OP pesticides in each cohort are shown in Table 3 (see also Figure S1). The geometric mean and median levels of total DAPs were highest in the agricultural CHAMACOS population, similar in the HOME study and Mount Sinai populations, and lowest in the Columbia cohort, despite the fact that the HOME study was the only study to enroll participants after the residential ban on chlorpyrifos (a DEP pesticide). The total DAP concentrations are dominated by DMP metabolites, with concentrations of DMP metabolites about three to four times greater than DEP metabolites.

**Table 3:** Distributions of urinary metabolites of organophosphate pesticides (nmol/g creatinine) measured during pregnancy, by cohort and pooled.

Cohort	*n*	Geometric Mean (SD)	< LOD (%)	Percentile				Maximum
				25th	50th	75th	95th	
CHAMACOS^*a*^
Total ΣDAP	484	105.63 (2.98)	0.0	50.3	102.0	220.9	697.0	3359.8
ΣDEP	484	16.41 (3.07)	0.0	7.8	16.3	34.8	100.6	453.5
ΣDMP	484	76.31 (3.63)	0.0	34.2	78.5	182.3	639.1	3228.9
HOME^*a*^
Total ΣDAP	328	66.78 (3.52)	1.6	28.9	70.1	154.2	466.8	1715.4
ΣDEP	328	11.15 (4.47)	16.9	4.5	14.5	31.8	89.3	199.1
ΣDMP	328	44.61 (4.28)	4.8	16.5	46.4	115.1	448.3	1686.8
Columbia
Total ΣDAP	82	42.88 (7.44)	7.3	21.3	59.6	135.3	638.0	1622.8
ΣDEP	82	7.39 (13.38)	19.5	4.9	19.4	36.1	110.4	231.8
ΣDMP	82	11.36 (20.63)	25.6	2.9	29.1	110.3	445.4	1584.2
Mount Sinai
Total ΣDAP	341	75.54 (5.50)	4.1	29.5	77.9	209.0	894.7	8498.6
ΣDEP	341	12.79 (8.96)	14.4	6.9	19.4	52.9	163.1	1629.5
ΣDMP	341	41.10 (7.58)	5.6	13.3	47.3	153.9	760.4	8498.5
Pooled
Total ΣDAP	1,235	80.30 (4.14)	2.1	37.1	87.1	199.4	753.1	8498.6
ΣDEP	1,235	13.11 (5.49)	10.2	6.2	16.6	37.5	113.9	1629.5
ΣDMP	1,235	49.15 (5.94)	4.7	20.6	57.2	149.5	631.6	8498.5
Abbreviations: ΣDAP, dialkyl phosphate metabolites; ΣDEP, diethyl phosphate metabolites; ΣDMP, dimethyl phosphate metabolites. ^***a***^CHAMACOS and HOME urinary metabolites are the average of two pregnancy measurements. Columbia and Mount Sinai are a single pregnancy measure.								

**Figure 1:**
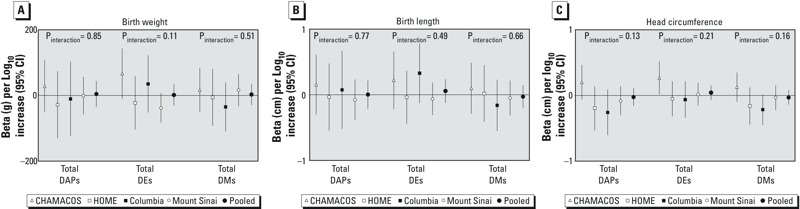
Association of 10-fold increase in maternal prenatal urinary total DAP, DMP, and DEP metabolites with infant birth weight (*A*), length (*B*), and head circumference (*C*) in each cohort and in the pooled data set. Models were adjusted for sex, race/ethnicity (non-Hispanic white, non-Hispanic black, Hispanic, other), country of origin (USA, other), marital status (married/living as married, single), maternal education (< high school, high school graduate), smoking during pregnancy, parity (nulliparous, multiparous), maternal age at delivery, and gestational age (spline). Interaction *p*-values from Wald tests on cross-product terms.

No associations were seen between total urinary ΣDAP, ΣDEP, or ΣDMP and birth weight, length, and head circumference in the pooled cohort (Table 4). Associations with 10-fold increases in urinary ΣDAP concentrations were close to null for birth weight [β = 4.47; 95% confidence interval (CI): –35.56, 44.49], length (β = –0.00; 95% CI: –0.21, 0.22), and head circumference (β = –0.02; 95% CI: –0.16, 0.11). Estimates were similar for 10-fold increase in ΣDEP and ΣDMP metabolites (Table 4). Interactions with cohort were not statistically significant for any of the exposures or outcomes (*p*-values ranged from 0.11 to 0.85; Figure 1). Estimates stratified by cohort (Table 4 and Figure 1) were similar but not identical to those of previously published reports (Eskenazi et al. 2004; Rauch et al. 2012; Wolff et al. 2007) in which the CHAMACOS cohort tended to show positive trends of urinary OP metabolites with fetal growth while the other cohorts showed negative or null associations. Differences from previously reported results were attributable to slight changes in exclusions, sample size, covariates, correction for urinary dilution, and substitution methods for values < LOD.

**Table 4:** Associations of 10-fold increases in urinary metabolites of organophosphate pesticides and fetal growth, by individual cohort and pooled data set.

Cohort, metabolite	Birth weight (g)			Length (cm)			Head circumference		
	*n*	β (95% CI)	*p*-Value	*n*	β (95% CI)	*p*-Value	*n*	β (95% CI)	*p*-Value
CHAMACOS
Total ΣDAP	484	28.06 (–51.35, 107.46)	0.49	478	0.15 (–0.30, 0.61)	0.51	468	0.20 (–0.06, 0.46)	0.13
ΣDEP	484	66.59 (–10.09, 143.26)	0.09	478	0.22 (–0.22, 0.66)	0.32	468	0.27 (0.01, 0.52)	0.04
ΣDMP	484	16.25 (–51.31, 83.82)	0.64	478	0.10 (–0.29, 0.49)	0.62	468	0.13 (–0.10, 0.35)	0.26
HOME
Total ΣDAP	307	–28.29 (–130.43, 73.85)	0.59	300	–0.03 (–0.55, 0.48)	0.89	301	–0.19 (–0.53, 0.14)	0.26
ΣDEP	307	–22.84 (–104.18, 58.50)	0.58	300	–0.04 (–0.44, 0.37)	0.85	301	–0.05 (–0.32, 0.22)	0.70
ΣDMP	307	–5.71 (–92.62, 81.19)	0.90	300	0.02 (–0.42, 0.45)	0.93	301	–0.16 (–0.45, 0.12)	0.27
Columbia
Total ΣDAP	82	–10.40 (–124.30, 103.50)	0.86	80	0.07 (–0.52, 0.67)	0.80	79	–0.26 (–0.61, 0.09)	0.14
ΣDEP	82	35.11 (–52.03, 122.26)	0.42	80	0.33 (–0.13, 0.78)	0.15	79	–0.06 (–0.34, 0.21)	0.65
ΣDMP	82	–34.87 (–109.57, 39.84)	0.35	80	–0.16 (–0.55, 0.23)	0.41	79	–0.22 (–0.45, 0.02)	0.07
Mount Sinai
Total ΣDAP	296	–0.65 (–58.07, 56.76)	0.98	294	–0.08 (–0.39, 0.23)	0.63	295	–0.08 (–0.30, 0.14)	0.47
ΣDEP	296	–37.96 (–83.11, 7.18)	0.10	294	–0.06 (–0.31, 0.18)	0.62	295	0.02 (–0.16, 0.19)	0.86
ΣDMP	296	16.21 (–32.73, 65.14)	0.52	294	–0.05 (–0.31, 0.22)	0.71	295	–0.03 (–0.22, 0.16)	0.74
Pooled
Total ΣDAP	1,169	4.47 (–35.56, 44.49)	0.83	1,152	0.00 (–0.21, 0.22)	0.98	1,143	–0.02 (–0.16, 0.11)	0.73
ΣDEP	1,169	1.55 (–31.58, 34.67)	0.93	1,152	0.06 (–0.12, 0.24)	0.53	1,143	0.04 (–0.07, 0.15)	0.46
ΣDMP	1,169	2.96 (–29.66, 35.58)	0.86	1,152	–0.03 (–0.21, 0.15)	0.75	1,143	–0.03 (–0.14, 0.08)	0.59
Abbreviations: ΣDAP, dialkyl phosphate metabolites; ΣDEP, diethyl phosphate metabolites; ΣDMP, dimethyl phosphate metabolites. All urinary metabolites were creatinine-corrected. CHAMACOS and HOME urinary metabolites are the average of two pregnancy measurements. Models were adjusted for center, sex, race/ethnicity (non-Hispanic white, non-Hispanic black, Hispanic, other), country of origin (USA, other), marital status (married/living as married, not married/living as married), maternal education (< and ≥ high school diploma/equivalent), smoking during pregnancy, parity (nulliparous and multiparous), maternal age at delivery, and gestational age (spline).									

**Figure 2:**
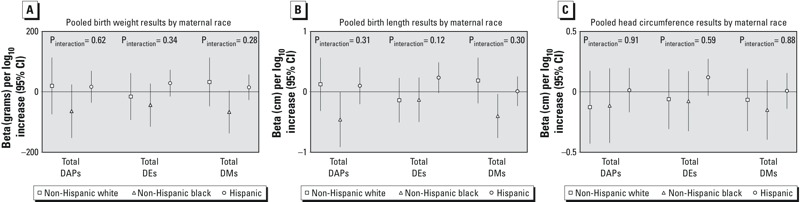
Association of 10-fold increase in maternal prenatal urinary total DAP, DMP, and DEP metabolites with infant birth weight (*A*), length (*B*), and head circumference (*C*) in the pooled data set, stratified by race/ethnicity. Models adjusted for cohort, sex, country of origin (USA, other), marital status (married/living as married, single), maternal education (< high school, high school graduate), smoking during pregnancy, parity (nulliparous, multiparous), maternal age at delivery, and gestational age (spline). Participants of “other” race/ethnicity not included in figure. Interaction *p*-values from Wald tests on cross-product terms.

**Figure 3:**
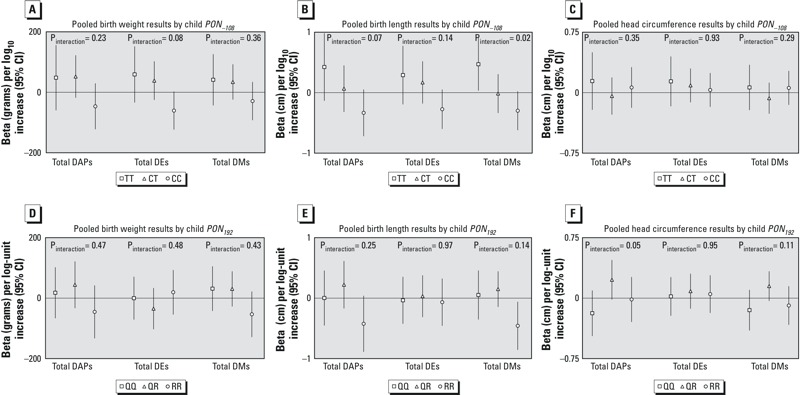
Association of 10-fold increase in maternal prenatal urinary total DAP, DMP, and DEP metabolites with infant birth weight, length, and head circumference in the pooled data set, stratified by infant *PON_–108_* genotype (*A*–*C*) and infant *PON_192_* genotype (*D*–*F*). Models adjusted for cohort, sex, race/ethnicity (non-Hispanic white, non-Hispanic black, Hispanic, other), country of origin (USA, other), marital status (married/living as married, single), maternal education (< high school, high school graduate), smoking during pregnancy, parity (nulliparous, multiparous), maternal age at delivery, and gestational age (spline). Interaction *p*-values from Wald tests on cross-product terms.

No statistically significant interaction (α = 0.1) was seen for race/ethnicity (Figure 2), sex (data not shown), or education (data not shown). Although interaction by race/ethnicity was not statistically significant, we present stratified results (Figure 2) for non-Hispanic white, non-Hispanic black, and Hispanic women (women of “other” race/ethnicity were dropped from the analysis due to small numbers) because the HOME study has reported differences in associations of DAP and birth outcome by race/ethnicity. We did find statistically significant interaction for some OP metabolites and *PON1* genotypes, so we present results stratified by child (Figure 3) and maternal (see Figure S2) *PON1* genotype.

In models stratified by race/ethnicity (Figure 2), higher concentrations of total ΣDMP were associated with lower infant weight (β = –66.78 g; 95% CI: –137.46, 3.90) and length (β = –0.40 cm; 95% CI: –0.76, –0.04), and ΣDAP were associated with decreased birth length (β = –0.46 cm; 95% CI: –0.91, –0.01) in non-Hispanic black women. When Hispanic blacks (specifically Dominican women in the Columbia cohort) were classified as black rather than Hispanic in sensitivity analyses, the associations between ΣDMP and birth weight (β = –43.63 g; 95% CI: –97.23, 9.97) and length (β = –0.23 cm; 95% CI: –0.51, 0.06) among blacks persisted but were attenuated, and negative associations were also seen with ΣDMP and head circumference (β = –0.18 cm; 95% CI: –0.37, 0.02), although none of the associations was statistically significant.

The distribution of maternal and child *PON1* SNPs differed among the four cohorts (Table 2) and was likely related to the differing racial/ethnic composition of the cohorts since *PON1* allele frequencies have been shown to vary by race/ethnicity (Rojas-Garcia et al. 2005). Analyses stratified by infant *PON1_–108_* genotype (Figure 3), revealed nonsignificant negative associations of birth weight (β = –60.53 g; 95% CI: –123.59, 2.53) and ΣDEP and of length and ΣDAP (β = –0.33 cm; 95% CI: –0.72, 0.05), ΣDEP (β = –0.27 cm; 95% CI: –0.60, 0.05), and ΣDMP (β = –0.29 cm; 95% CI: –0.62, 0.02) among infants with the *PON1_–108CC_* genotype. In contrast, positive associations of DAPs and birth outcomes were seen among infants with the *PON1_–108TT_* genotype, including a statistically significant positive association of ΣDMP and length (β = 0.47 cm; 95% CI: 0.03, 0.91). Interaction terms for genotype × DAPs were statistically significant in the models of ΣDEP on birth weight (*p* = 0.08) and ΣDAP and ΣDMP on length (*p* = 0.07 and *p* = 0.02, respectively). With infant *PON1_192_* genotype, we observed negative associations of length and ΣDAP (β = –0.42 cm; 95% CI: –0.89, 0.04; *p*_interaction_ = 0.25) and ΣDMP (β = –0.46 cm; 95% CI: –0.86, –0.06; *p*_interaction_ = 0.14) among the *RR* group. Positive associations were seen with head circumference and ΣDAP (β = –0.23 cm; 95% CI: –0.02, 0.47; *p*_interaction_ = 0.05) and ΣDMP (β = –0.15 cm; 95% CI: –0.03, 0.33; *p*_interaction_ = 0.11) among the heterozygous *QR* group. No statistically significant associations were seen for DAPs and birth outcome stratifying by maternal *PON1* genotype (see Figure S2), except for a positive association of total ΣDEP and head circumference (β = 0.31 cm; 95% CI: 0.07, 0.55; *p*_interaction_ = 0.13) in mothers with the *PON1_192QR_* genotype. Interaction terms for genotype × DAPs were statistically significant in the models of ΣDAP and ΣDMP on head circumference (*p* = 0.09 for both), again with positive associations seen in the heterozygous *PON1_192QR_* genotype, although no associations were statistically significant. Maternal *PON1_192_* genotype was not available for Columbia participants, and no maternal *PON1* genotypes were available for HOME participants.

In sensitivity analyses, results did not change substantively when the sample was limited to primiparous women (*n* = 638) or term births (*n* = 1,090) or when women with pregnancy complications (*n* = 103) were included (data not shown). Results were also similar when the DAP concentrations were uncorrected for creatinine, when values < LOD were assigned LOD divided by the square root of 2, and when only one late pregnancy measure of urinary metabolites was used for each woman (data not shown).

## Discussion

We found no significant associations between metabolites of OP pesticides and birth weight, length, and head circumference in a pooled data set of > 1,000 pregnant women from four birth cohort studies. Animal studies suggest that OPs may restrict fetal growth, possibly by influencing the adenylyl cyclase signaling cascade, by increasing thyroxine levels, or by effects on placental transport of nutrients (Eskenazi et al. 1999). However, previously published results from these four birth cohorts (Eskenazi et al. 2004; Rauch et al. 2012; Whyatt et al. 2004; Wolff et al. 2007) showed associations between OP exposure and birth outcome that were inconsistent across studies. However, we did observe inverse associations of DAP and DM metabolites with birth length and nonsignificant inverse associations of DM metabolites with birth weight among black women. This is consistent with findings of decreased birth weight with increasing DAP, DE, and DM concentrations among black women in the HOME study (Rauch et al. 2012) and decreased birth weight and length with increasing blood concentrations of chlorpyrifos in African-American and Dominican women in the Columbia study (Whyatt et al. 2004)—although it should be noted that chlorpyrifos devolves to DEP metabolites (Bravo et al. 2002), and the main association seen in blacks in this study was with DMPs.

We hypothesized that we might see inverse associations of urinary OP metabolites with birth outcomes in mothers or children with the more vulnerable *PON1_–108TT_* and *PON1_192QQ_* genotypes. In the Mount Sinai cohort, DEP metabolites were associated with significantly lower birth weight among mothers with the *PON1_192QQ_* genotype (Wolff et al. 2007). In the CHAMACOS study, the inverse associations of DAPs with gestational duration were strongest among infants with *PON1_–108TT_* and *PON1_192QQ_* genotypes (Harley et al. 2011). However, this pattern was not seen in the HOME cohort, where decrements in birth weight and gestational age associated with DAP were greatest among infants with heterozygous *PON1_–108CT_* and *PON1_192QR_* genotypes (Rauch et al. 2012). In the pooled analysis, birth weight was negatively associated with total DEPs in infants with the *PON1*_–108_ *_CC_* genotype, and birth length was negatively associated with both DAP and DMP metabolites among infants with the *PON1*_–108_ *_CC_* and *PON1*_192_ *_RR_* genotypes, rather than the less active *PON1*_–108_ *_TT_* and *PON1*_192_ *_QQ_* genotypes, as hypothesized. We found limited interaction of DAPs with maternal *PON1* genotype, and the patterns of association did not support stronger negative associations among women with the more susceptible genotypes, even though maternal genotype might be hypothesized to be the more powerful determinant of susceptibility than infant genotype given that mothers have approximately four times the paraoxonase enzyme as newborns and have a 50% overlap with infant genotype (Holland et al. 2006). We had fewer observations for the maternal genotype analysis because HOME study participants did not have maternal genotype data for either SNP, and Columbia study participants had data for only one; however, there was little or no evidence of consistent patterns of association, as might be expected if the only issue was reduced precision. Thus, it is possible that the patterns with infant genotype were attributable to chance. An additional issue is that *PON1* allele frequencies vary by race/ethnicity, as can be seen in the different SNP distributions across cohorts. Because race/ethnicity is associated with birth outcome and *PON1* genotype and may also be associated with OP exposure, confounding by race/ethnicity is a possibility. Although we controlled for race/ethnicity in the models, there is considerable genetic variability within race/ethnicity categories (perhaps especially among the Hispanic category, which included women of Mexican, Puerto Rican, and Dominican origin), suggesting that we cannot completely discount the possibility of residual confounding.

In addition to racial and ethnic differences, the cohorts also varied in sources and routes of OP pesticide exposures, including food, home pesticide use, and agricultural drift or take-home exposure. Thus, urinary DAP concentrations may reflect different exposures in each cohort, limiting their interpretation in the pooled data set. In the farmworker CHAMACOS population, urinary DAPs likely represented a combination of agricultural, residential, and dietary exposure, a different mix of pesticides than would be experienced by the low-income, urban Columbia and Mount Sinai populations who were presumably exposed only through residential and dietary routes, or the more affluent HOME study participants who were enrolled after the residential ban and likely exposed mainly through diet. The HOME participants had relatively high DAP levels considering that their exposure was mainly dietary, suggesting that they may have had higher fruit and vegetable consumption, a very different route of exposure than women who may be exposed from home pesticide use.

Not all of the six urinary DAPs were equally detected. The proportion of women with values below the detection limit in the pooled sample ranged from 9% for DMTP to 78% for DEDTP. Individual OPs do not necessarily devolve to all three DE or DM metabolites—for example, malathion devolves to DMP, DMTP (dimethylthiophosphate), and DMDTP (dimethyldithiophosphate), whereas oxydemeton-methyl devolves only to DMP and DMTP (Bravo et al. 2002)—and it is expected that certain metabolites have lower detection frequencies than others. For this reason, we examined ΣDEP and ΣDMP metabolites to obtain comprehensive estimates of exposure to all DE- and DM-devolving OP pesticides. However, the large number of women with concentrations < LOD for some metabolites may have resulted in minor misclassification at the very lowest exposure levels. Misclassification of these minimal exposures would not be expected to seriously bias the exposure–response estimation across the full range of exposure.

Although urinary DAPs are a useful biomarker of OP pesticide exposure, they have limitations. OPs have short half-lives in the body and show considerable intra-individual variability over time; thus, urinary DAPs represent short-term rather than ongoing exposure. DAPs are nonspecific metabolites, and not all pesticides that devolve to the same metabolite are equally toxic; for example, both oxydemeton-methyl and malathion devolve to DMP metabolites, but the former is much more toxic (U.S. EPA 2002). Although we can determine whether the parent pesticide was from the DEP or DMP class, we cannot determine the mix of actual pesticide parent compounds to which the mother was exposed. Thus, we cannot examine how the toxicity of the pesticide mixture differed across cohorts. Additionally, OPs can break down to DAPs in the environment as well as in the body, so DAP concentrations in urine reflect a mixture of exposure to toxic parent compounds and nontoxic preformed DAPs in dust and food (Quirós-Alcalá et al. 2012). The proportion of preformed DAPs may be greater in populations receiving their exposure predominantly from food, where the parent pesticide has had more time to break down into DAPs before consumption. Finally, although DAP concentrations were all measured in the same laboratory using the same analytical methods, samples were stored for different amounts of time and analysis took place in different years. Thus, we cannot rule out the possibility of storage or batch effects, suggesting that, although rank order of DAP concentrations is internally consistent within cohort, rank may not be accurate when DAP concentrations measured in different batches (i.e. cohorts) are pooled together.

One strength of this study is its large sample size, which allows for better examination of race/ethnicity and genetic variability than in the more homogenous, individual cohorts. Methodologies were similar between cohorts and tests of interaction by cohort failed to reject the hypothesis of homogeneity by cohort. Analyses in our pooled cohort confirmed earlier findings of associations of OP exposure (as measured by DAPs in urine and chlorpyrifos in blood) with decreased birth weight in non-Hispanic black women, but not in whites or Hispanics (Rauch et al. 2012; Whyatt et al. 2004). In general, non-Hispanic black women had a wider range and more variability in birth weight, which may explain why an association was seen in this group.

Overall, this study does not support associations of OP pesticide exposure during pregnancy with decreased weight, length, or head circumference at birth, except in black women and possibly infants with the *PON1_–108CC_* and *PON1_192RR_* genotypes. However, this study also illustrates the limitations of pooling data when the population demographics and exposure patterns for biomarkers of exposure with a short half-life differ across cohorts. Although pooling data from multiple cohorts is tempting, careful consideration must be given to the interpretation of the exposure measures in each cohort and whether rank order is maintained and remains meaningful across the pooled cohort. In some cases, individual cohort studies with homogeneous populations may be preferable to pooled data.

**Editor’s Note:** In the Advance Publication, the symbols in the figure legends for Figures 2 and 3 were incorrect. In the Figure 2A–C legends, the correct symbols are triangles to indicate “Non-Hispanic Black” and circles to indicate “Hispanic.” In the Figure 3A–C legends, the correct symbols are triangles to indicate “CT” and circles to indicate “CC.” In the Figure 3D–F legends, the correct symbols are triangles to indicate “QR” and circles to indicate “RR.” The correct figure legends are included in this article.

## Supplemental Material

(749 KB) PDF
