# Supplementary material for: Prenatal Exposure to Organophosphorous Pesticides and Fetal Growth: Pooled Results from Four Longitudinal Birth Cohort Studies
Source: Environ Health Perspect. 2015 Dec 18;124(7):1084–92. doi: 10.1289/ehp.1409362 (PMC4937849; doi:10.1289/ehp.1409362)
Supplement: (749 KB) PDF [file ehp.1409362.s001.acco.pdf]

Environ Health Perspect
DOI: 10.1289/ehp.1409362

Editor's Note: *In the Supplemental Material, the symbols in the figure legends of Figure S2 were incorrect. In the Figure S2A–C legends the correct symbols are triangles to indicate "CT" and circles to indicate "CC." In the Figure S2D–F legends, the correct symbols are triangles to indicate "QR" and circles to indicate "RR." The figure legends have been corrected in this PDF.*

Note to readers with disabilities: *EHP* strives to ensure that all journal content is accessible to all readers. However, some figures and Supplemental Material published in *EHP* articles may not conform to 508 standards due to the complexity of the information being presented. If you need assistance accessing journal content, please contact ehp508@niehs.nih.gov. Our staff will work with you to assess and meet your accessibility needs within 3 working days.

Supplemental Material

Prenatal Exposure to Organophosphorous Pesticides and Fetal Growth: Pooled Results from Four Longitudinal Birth Cohort Studies

Kim G. Harley, Stephanie M. Engel, Michelle G. Vedar, Brenda Eskenazi, Robin M. Whyatt, Bruce P. Lanphear, Asa Bradman, Virginia A. Rauh, Kimberly Yolton, Richard W. Hornung, James G. Wetmur, Jia Chen, Nina T. Holland, Dana Boyd Barr, Frederica P. Perera, and Mary S. Wolff

Table of Contents

Table S1: Limits of detection (LOD) and percent below LOD for each of the 6 dialkyl phosphate urinary metabolites measured at each Center.

Figure S1. Urinary metabolites of organophosphate pesticides (nmol/g creatinine) measured during pregnancy by cohort showing ΣDAPs (A), ΣDEPs (B), and ΣDMPs (C).

Figure S2. Association of 10-fold increases in maternal prenatal urinary DAP, DM, and DE metabolites with infant birth weight, length, and head circumference in the pooled dataset, stratified by maternal PON-108 genotype (A-C) and maternal PON192 genotype (D-F).

Table S1: Limits of detection (LOD) and percent below LOD for each of the 6 dialkyl phosphate urinary metabolites measured at each Center.

	CHAMACOS		HOME		Columbia		Mount Sinai		Total
**Metabolite**	**LOD**	**<LOD (%)**	**LOD**	**<LOD (%)**	**LOD**	**<LOD (%)**	**LOD**	**<LOD (%)**	**<LOD (%)**
Diethylphosphate (DEP)	0.2	40.7%	0.6	40.8%	0.3	43.9%	0.3	61.9%	47.0%
Diethylthiophosphate (DETP)	0.1	0.7%	0.4	37.6%	0.4	32.9%	0.4	20.8%	18.5%
Diethyldithiophosphate (DEDTP)	0.1	71.1%	0.4	80.3%	0.1	56.1%	0.2	91.8%	78.4%
Dimethylphosphate (DMP)	0.6	4.7%	0.6	44.0%	0.5	42.7%	0.5	48.1%	30.2%
Dimethylthiophosphate (DMTP)	0.2	1.1%	0.2	9.9%	0.7	46.3%	0.4	10.0%	9.1%
Dimethyldithiophosphate (DMDTP)	0.1	42.2%	0.5	54.5%	0.2	70.7%	0.3	75.4%	57.0%
All 3 DEs <LOD		0.0%		16.9%		19.5%		14.4%	10.2%
At least 1 DE <LOD		80.2%		88.9%		72.0%		94.4%	86.0%
All 3 DMs <LOD		0.0%		4.8%		25.6%		5.6%	4.7%
At least 1 DM <LOD		45.1%		72.9%		79.3%		83.9%	66.0%
All 6 DAPs <LOD		0.0%		1.6%		7.3%		4.1%	2.1%
At least 3 DAPs <LOD		18.5%		54.8%		57.3%		65.7%	44.1%
At least 1 DAP <LOD		88.4%		95.5%		90.2%		97.7%	93.1%

Figure S1. Urinary metabolites of organophosphate pesticides (nmol/g creatinine) measured during pregnancy by cohort showing ΣDAPs (A), ΣDEPs (B), and ΣDMPs (C).

A. **B.** **C.**

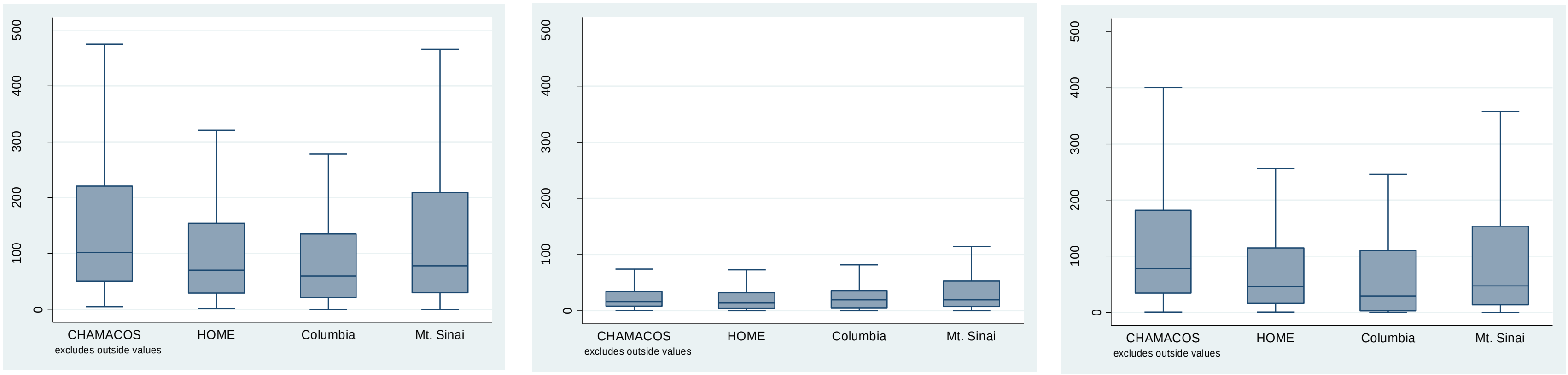

The 25th, 50th, and 75th percentiles are shown by the box. The upper whisker represents 1.5 times the upper IQR (top portion of the box), with values above the top of the whisker not shown ("excludes outside values"). The lower whisker represents 1.5 times the lower IQR, stopping at the lowest value reported.

Figure S2. Association of 10-fold increases in maternal prenatal urinary DAP, DM, and DE metabolites with infant birth weight, length, and head circumference in the pooled dataset, stratified by maternal PON-108 genotype (A-C) and maternal PON192 genotype (D-F).

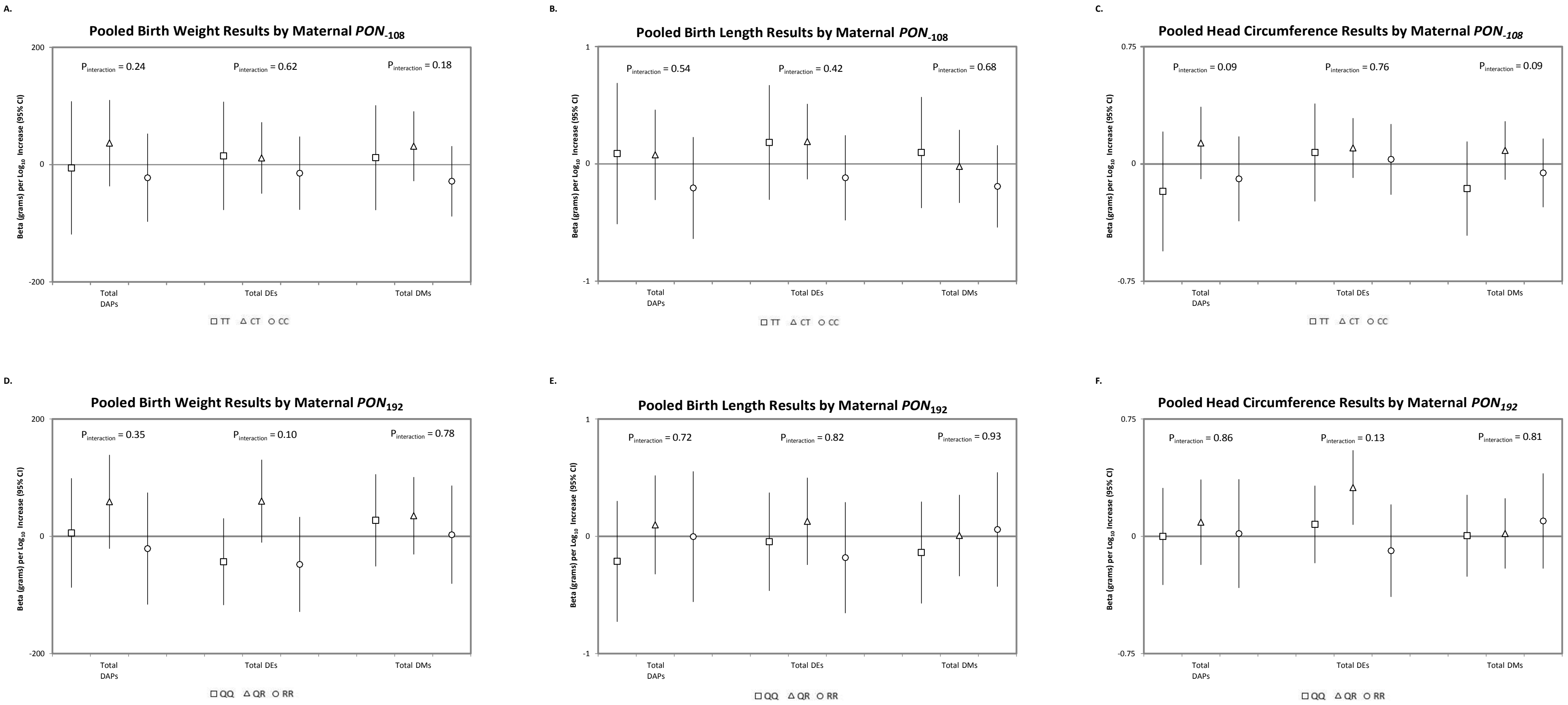

Models adjusted for cohort, sex, country of origin (US, Other), marital status (married/living as married, single), maternal education (< high school, high school graduate), smoking during pregnancy, parity (nulliparous, multiparous), maternal age at delivery, and gestational age (spline). Maternal PON1-108 and PON1192 genotype was not available for HOME study participants, maternal PON1192 genotype was not available for Columbia participants. Interaction p-values from Wald tests on cross-product terms.
